# Influenza colloidal gold method and blood routine tests combination for rapid diagnosis of influenza: a decision tree-based analysis

**DOI:** 10.1038/s41533-021-00251-x

**Published:** 2021-07-15

**Authors:** Xiaoguang Li, Jing Chen, Fei Lin, Wei Wang, Jie Xu, Nan Li

**Affiliations:** 1 Department of Infectious Diseases, Peking University Third Hospital, Beijing, People’s Republic of China; 2 Research Center of Clinical Epidemiology, Peking University Third Hospital, Beijing, People’s Republic of China

**Keywords:** Population screening, Physical examination

## Abstract

Rapid influenza diagnosis can facilitate targeted treatment and reduce antibiotic misuse. However, diagnosis efficacy remains unclear. This study examined the efficacy of a colloidal gold rapid test for rapid influenza diagnosis. Clinical characteristics of 520 patients with influenza-like illness presenting at a fever outpatient clinic during two influenza seasons (2017–2018; 2018–2019) were evaluated. The clinical manifestations and results of routine blood, colloidal gold, and nucleic acid tests were used to construct a decision tree with three layers, nine nodes, and five terminal nodes. The combined positive predictive value of a positive colloidal gold test result and monocyte level within 10.95–12.55% was 88.2%. The combined negative predictive value of a negative colloidal gold test result and white blood cell count > 9.075 × 10^9^/L was 84.9%. The decision-tree model showed the satisfactory accuracy of an early influenza diagnosis based on colloidal gold and routine blood test results.

## Introduction

Seasonal influenza is an acute viral infection that affects people of all age groups worldwide. According to World Health Organization (WHO) estimates, influenza viruses infect between 5 and 15% of the global population, causing an estimated 3–5 million severe cases and up to 650,000 respiratory deaths a year^1,2^. There is no influenza-specific treatment, although antiviral drugs and supportive treatments are used to alleviate discomfort, shorten the disease course, and reduce the mortality risk. However, in some countries and regions, the lack of accurate and rapid influenza diagnostics has resulted in widespread misuse of antibiotics administered to patients while awaiting an influenza diagnosis.

Craddock et al.^3^ reported that 17.2 and 25.4% of viral acute upper respiratory tract infections (AURTI) were inappropriately treated with antibiotics at urban internal medicine (IM) and family medicine (FM) ambulatory care clinics, respectively. A previous study reported that, among 6136 patients with acute respiratory infections (ARIs), 2522 (41%) had diagnoses for which antibiotics are not indicated; moreover, 2106 (84%) patients were diagnosed as having a viral upper respiratory tract infection or bronchitis^4^. Another study showed that 40% of upper respiratory tract infections (URTI) were treated with antibiotics in the post-pandemic influenza period^5^. Furthermore, antibiotic prescriptions from deidentified administrative claims data reached 9.8 million in 2013–2015, and antibiotics prescribed to 3.9 million insurance beneficiaries were used mainly for respiratory tract infections, such as the common cold and seasonal flu^6^. This evidence suggests that the ability to distinguish cases of influenza from influenza-like diseases can improve the efficacy of aggressive treatment approaches and reduce antibiotic misuse.

Eboigbodin et al.^7^ showed that the present rapid influenza diagnostic methods, including onsite rapid nucleic acid tests, allow prompt treatment of patients and limit the inappropriate use of antibiotics and antiviral drugs, although they are expensive. The combined use of a conventional colloidal gold test with routine blood tests and the clinical symptoms has some efficacy in influenza diagnosis despite a low overall performance^8^. Antibiotic misuse is a global problem, and regional antibiotic misuse may increase the prevalence of drug-resistant bacteria contributing to the global risk of infectious diseases^9^. Considering these challenges, developing an effective and economical rapid influenza diagnosis method is of paramount importance.

Studies have previously used statistical methods to construct an accurate disease, diagnostic model. These proposed models were based on patient characteristics and intended to leverage known markers for the diagnosis. The Classification Regression Tree (CRT)-based decision tree models are considered representative of clinical diagnosis, and their conclusions are easy to communicate and applicable in clinical practice. Recently, decision tree-based diagnostic models have been increasingly used in various disciplines. In influenza-related research, nucleic acid test results are used as a diagnostic gold standard reference for colloidal gold tests, with the clinical presentation and results of routine blood tests. The primary objective of this study was to establish a rapid diagnostic model for influenza patients based on colloid gold tests and clinical characteristics.

## Results

Table 1 presents the clinical presentation characteristics of 520 patients with influenza-like illness.

**Table 1:** Clinical presentations of 520 patients with influenza-like illness.

Type	Item	Mean ± SD/*n* (%)
Demographics	Age (years)	34.82 ± 14.06
	16–19	40 (7.7%)
	20–39	345 (66.4)
	40–64	108 (20.7)
	≥65	27 (5.2%)
	Male	249 (47.9%)
	Female	271 (52.1%)
Disease course, days	1	263 (50.6%)
	2	121 (23.3%)
	3	136 (26.1%)
Clinical presentation	Body temperature (°C)	38.71 ± 0.53
	38–38.9 °C	337 (64.8%)
	≥39 °C	183 (35.2%)
	Cough	322 (61.9%)
	Sputum	155 (29.8%)
	Dyspnea	14 (2.7%)
	Runny nose	163 (31.4%)
	Diarrhea	13 (2.5%)
	Dizziness and headache	297 (57.1%)
	Myalgia and arthralgia	276 (53.1%)
	Fatigue	256 (49.2%)
	Chills	154 (29.6%)
Medical history	Underlying disease	10 (1.9%)
	Pregnancy	4 (0.8%)
	Contact with infected patients	62 (11.9%)
	Influenza vaccination	0 (0%)
	High-risk population^a^	38 (7.3%)
Laboratory examinations	Influenza colloidal gold test	424 (81.5%)^b^
	Influenza A^b^	174 (33.5%)
	Influenza B	63 (12.11%)
	Chest X-ray	83 (16.0%)
	Pneumonia	32 (6.2%)
	Blood routine	490 (94.2%)
	White blood cell count, ×10^9^/L	7.69 ± 3.69
	WBC > 10 × 10^9^/L	100 (19.2%)
	Hemoglobin level, g/L	143.98 ± 15.30
	Platelet count, × 10^12^/L	193.69 ± 50.48
	Neutrophil %	68.82 ± 14.21
	>75%	168 (32.3%)
	Lymphocyte %	18.67 ± 10.36
	<20%	276 (53.1%)
	Monocyte %	10.81 ± 3.72
	>10%	262 (50.4%)

^a^A population was considered “high-risk” if it had any of the following characteristics: age >65 years, an underlying disease or immunodeficiency, or pregnancy.

^b^Two patients who underwent the colloidal gold test simultaneously tested positive for influenza A and B.

### Correlation between clinical presentation and nucleic acid test results

A total of 520 patients were divided into two groups based on the results of the nucleic acid test: 271 with “positive” nucleic acid test results and 249 patients with “negative” nucleic acid test results. Among the 271 patients with positive nucleic acid test results, there were 116 cases of H1N1 (2009) influenza A, 55 cases of seasonal influenza H3, 69 cases of Yamagata (BY) influenza B, and 31 cases of Victoria (BV) influenza B. The sex and age were unassociated with nucleic acid test results. Patients with a positive nucleic acid test result had a slightly longer disease course (*t* = 2.429, *P* = 0.015). The average body temperature of both groups was similar (38.75 ± 0.52 vs. 38.67 ± 0.54, *t* = 1.656, *P* = 0.098). The positive group had a higher proportion of patients with cough, runny nose, and chills than in the negative group (*χ*^2^ = 7.540, 10.412, and 8.035, respectively; *P* = 0.006, 0.001, and 0.005, respectively). In the positive group, a higher number of patients underwent a colloidal gold test than in the negative group (*χ*^2^ = 14.848, *P* < 0.001). Furthermore, patients in the positive group had a lower WBC count and neutrophil percentage, and higher lymphocyte percentage and monocyte percentage than patients in the negative group (*t* = −4.256, −2.140, 2.004, and 2.836, respectively; *P* < 0.001, 0.033, 0.046, and 0.005, respectively; Table 2).

**Table 2:** Correlation between clinical characteristics and influenza diagnosis.

Characteristic (*N*)	Positive nucleic acid test result	Negative nucleic acid test result	*t*/*χ*^2^	*P* value
*N* = 520	271	249		
Age (years)	34.77 ± 14.14	33.12 ± 14.01	−0.082	0.935
16–19	22 (8.1%)	18 (7.2%)	0.191	0.979
20–39	178 (65.7%)	167 (67.1%)		
40–64	57 (21.0%)	51 (20.5%)		
>65	14 (5.2%)	13 (5.2%)		
Sex: male (249)	128 (51.4%)	121 (48.6%)	0.096	0.756
female (271)	143 (52.8%)	128 (47.2%)		
Disease course (days)	1.84 ± 0.05	1.66 ± 0.05	2.429	**0.015**
1 (263)	123 (46.8%)	140 (53.2%)	6.274	**0.043**
2 (121)	68 (56.2%)	53 (43.8%)		
3 (136)	80 (58.8%)	56 (41.2%)		
Body temperature (°C)	38.75 ± 0.52	38.67 ± 0.54	1.656	0.098
38–38.9 °C (337)	172 (51.0%)	165 (49.0%)	0.445	0.505
≥39 °C (183)	99 (54.1%)	84 (45.9%)		
Sore throat (349)	175 (50.1%)	174 (49.9%)	1.654	0.198
Cough (322)	183 (56.8%)	139 (43.2%)	7.540	**0.006**
Sputum (155)	91 (58.7%)	64 (41.3%)	3.848	**0.050**
Dyspnea (14)	10 (71.4%)	4 (28.6%)	1.429	0.232
Runny nose (163)	102 (62.6%)	61 (37.4%)	10.412	**0.001**
Diarrhea (13)	6 (46.2%)	7 (53.8%)	0.190	0.663
Dizziness and headache (297)	154 (51.9%)	143 (48.1%)	0.019	0.890
Myalgia and arthralgia (276)	149 (54.0%)	127 (46.0%)	0.824	0.364
Fatigue (256)	129 (50.4%)	127 (49.6%)	0.601	0.438
Chills (154)	95 (61.7%)	59 (38.3%)	8.035	**0.005**
Underlying disease (10)	4 (40.0%)	6 (60.0%)	0.207	0.649
Pregnancy (4)	3 (75.0%)	1 (25.0%)	0.171	0.679
Contact with infected patients (62)	35 (56.5%)	27 (43.5%)	0.530	0.466
Influenza vaccination (0)	0	0		
High-risk population^a^ (38)	20 (52.6%)	18 (47.4%)	0.004	0.947
Underwent colloidal gold test (424)	174 (74.041%)	61 (25.96%)	14.848	**<0.001**
Influenza A test (174)	126 (72.4%)	48 (27.6%)	31.770	**<0.001**
Influenza B test (63)	49 (77.8%)	14 (22.2%)	14.079	**<0.001**
Chest X-ray (83)	38 (45.8%)	45 (54.2%)	1.587	0.208
Pneumonia (32)	14 (43.8%)	18 (56.2)	0.956	0.328
Routine blood test (490)	253 (51.6%)	237 (48.4%)	0.793	0.373
White blood cell count (WBC), ×10^9^/L	6.97 ± 3.33	8.36 ± 3.90	−4.256	**<0.001**
WBC < 10×10^9^/L(390)	227 (58.2%)	163 (41.8%)	33.055	**<0.001**
Hemoglobin level, g/L	144.98 ± 14.67	143.59 ± 16.17	0.977	0.329
Platelet count, ×10^12^/L	190.82 ± 48.53	199.19 ± 59.62	−1.75	0.081
Neutrophil %	67.43 ± 14.22	70.23 ± 14.58	−2.140	**0.033**
<75% (322)	182 (56.5%)	140 (43.5%)	8.989	**0.003**
Lymphocyte %	19.81 ± 11.60	17.68 ± 10.22	2.004	**0.046**
<20% (276)	130 (47.1%)	146 (52.9%)	5.196	**0.023**
Monocyte %	11.28 ± 3.49	10.26 ± 3.98	2.836	**0.005**
>10% (262)	160 (61.1%)	102 (38.9%)	20.075	**<0.001**

^a^A population was considered “high-risk” if it had any of the following characteristics: age >65 years, an underlying disease or immunodeficiency, or pregnancy.

### Diagnostic performance of clinical characteristics associated with a definitive diagnosis of influenza

Nucleic acid testing of both positive and negative groups, showed differences in clinical characteristics, mainly including disease course, cough and expectoration, runny nose, chills, colloidal test, routine blood test white blood cell count, neutrophils %, lymphocytes %, monocytes %, with confirmed influenza nucleic acid detection. Further analysis showed the clinical characteristics in the confirmed diagnosis efficacy (Table 3).

**Table 3:** Diagnostic performance of clinical characteristics associated with a definitive diagnosis of influenza.

Characteristic	Sensitivity	Specificity	Positive predictive value	Negative predictive value	AUC (95% CI)
*N* = 520					
Disease course					
≥1 day	45.4%	43.8%	46.8%	42.4%	
Cough	67.5%	44.2%	56.8%	55.6%	
Productive cough	33.6%	74.3%	58.7%	50.7%	
Runny nose	37.6%	75.5%	62.6%	52.7%	
Chills	35.1%	76.3%	61.7%	51.9%	
Colloidal test (*n* = 424)	73.1%	67.2%	74.0%	66.1%	
Influenza A test	52.9%	74.2%	72.4%	55.2%	
Influenza B test	20.6%	92.5%	77.8%	47.7%	
Routine blood tests, white blood cell count (WBC) (*n* = 490)					0.604 (0.553–0.656)
WBC < 10 × 10^9^/L	89.7%	31.2%	58.2%	74.0%	
Neutrophil %					0.570 (0.519–0.621)
<75%	71.9%	40.9%	56.5%	57.7%	
Lymphocyte %					0.557 (0.502–0.612)
<20%	51.4%	38.4%	47.1%	42.5%	
Monocyte %					0.608 (0.554–0.662)
>10%	63.2%	57.0%	61.1%	59.2%	

### Decision tree-based examination of possible paths for further influenza screening

**Fig. 1:**
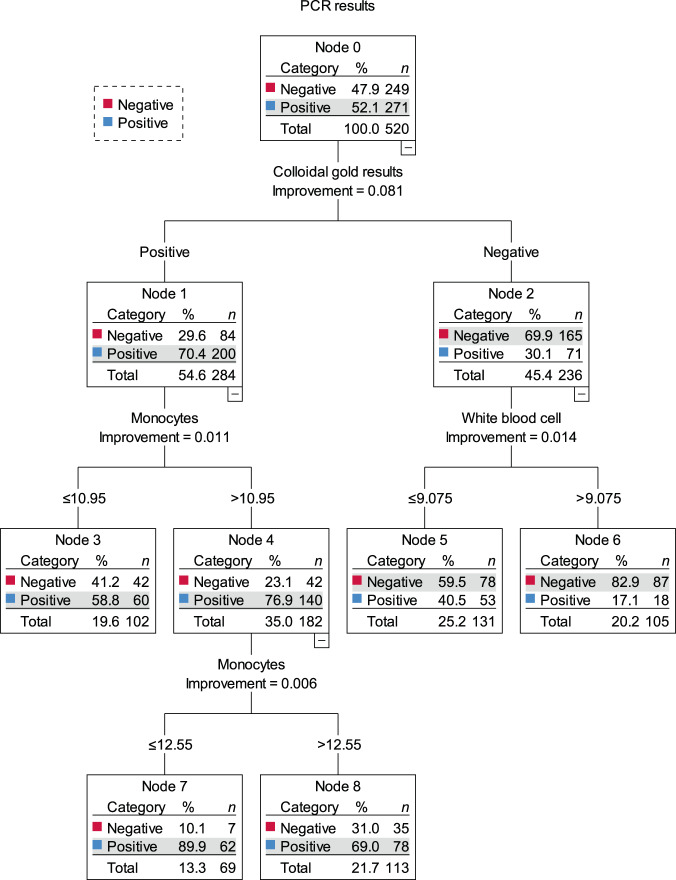
Results of the influenza diagnostic decision tree analysis. The CRT algorithm was used to construct a decision tree that had three layers, nine nodes, and five terminal nodes.

A positive nucleic acid test result was used as the gold standard for the diagnosis of influenza, and the colloidal gold test, clinical presentation, and routine blood test results were used as independent variables. The CRT algorithm was used to construct a decision tree that had three layers, nine nodes, and five terminal nodes (Fig. 1). There were two main decision cues. Patients with influenza-like illness (*n* = 520) were classified according to the value of the colloidal gold test node. Of the 236 patients with a negative colloidal gold test result, 165 had a negative nucleic acid test result, yielding a negative predictive value (NPV) of 69.9% for the colloidal gold method. Patients with a negative colloidal gold test result were further classified based on their WBC count: of the 105 patients with WBC count > 9.075 × 10^9^/L, 87 had a negative nucleic acid test result, implying that the NPV of this characteristic was 82.9%, which made it a decision cue candidate. The NPV of the colloidal gold method with WBC count > 9.075 × 10^9^/L was 82.9%.

Subsequently, 520 patients with influenza-like illness were classified according to the colloidal gold test results, and 284 patients with a positive colloidal gold test result were further classified according to the monocyte count. Thus, 140 out of 182 patients with the monocyte percentage > 10.95% had positive nucleic acid test results; the positive predictive value (PPV) was 76.9%. Moreover, among 140 patients, when the monocyte percentage was < 12.55% was used for classification, 62 out of 69 patients who met this criterion had a positive nucleic acid test result, and the PPV was 89.9%. These results indicate that these factors are potential candidates for decision cues. The PPV of the colloidal gold method with monocyte percentage within 10.95–12.55% was 89.9%. These factors are important contributors toward a definitive diagnosis of influenza.

### Verification of influenza screening pathway in the decision tree

In the 2019–2020 influenza season, 156 influenza-like illness cases were clinically diagnosed according to the decision tree before the nucleic acid test of pharyngeal swabs was performed. The nucleic acid test results of the 156 cases were as follows: 75 were positive (53 cases of influenza A H3, 6 cases of 2009 H11 influenza A, 16 cases of influenza B Victoria) and 81 were negative. According to the screening path of the decision tree analysis, the NPV of the node 2 colloidal gold test was 68.6%, and that of the node 6 colloidal gold test with negative results combined with WBC > 9.075 × 10^9^/L increased to 81.0%. The PPV of the node 4 colloidal gold test combined with mononuclear cells > 10.95% was 88.6%, while that of the node 7 colloidal gold test combined with mononuclear cells within 10.95–12.55% increased to 90.9%. These results were consistent with those of the decision tree (Table 4).

**Table 4:** Verification results of the decision tree of 156 influenza-like illness cases in 2019–2020.

Node	Category	Case	PCR verification	*N*	%
1	Colloidal gold test +	51	+	42	82.4%
			–	9	17.6%
2	Colloidal gold test –	105	+	33	31.4%
			–	72	68.6%
3	Colloidal gold test + monocytes ≤ 10.95%	16	+	11	68.7%
			–	5	31.3%
4	Colloidal gold test + monocytes > 10.95%	35	+	31	88.6%
			–	4	11.4%
5	Colloidal gold test − white blood cells ≤ 9.075 × 10^9^/L	63	+	25	39.7%
			–	38	60.3%
6	Colloidal gold test − white blood cells > 9.075 × 10^9^/L	42	+	8	19.0%
			–	34	81.0%
7	Colloidal gold test + monocytes 10.95–12.55%	11	+	10	90.9%
			–	1	9.1%
8	Colloidal gold test + monocytes > 12.55%	24	+	21	87.5%
			–	3	12.5%

## Discussion

This study included patients from the 2017–2018 and 2018–2019 influenza seasons and used data from the 2019–2020 influenza season to verify the results. The results of the decision tree analysis revealed that clinical symptoms did not determine the diagnosis of influenza. However, differentiating the clinical presentation of influenza from that of influenza-like disease is challenging. Therefore, the use of the colloidal gold test and routine blood tests, including WBC and monocyte count are key factors for an accurate influenza diagnosis. This study used decision tree analysis to show that a colloidal gold test and routine blood tests can increase the accuracy of the rapid diagnosis of influenza. The PPV of a positive result on the colloidal gold test combined with a monocyte percentage within 10.95–12.55% was 88.2%. The NPV of the negative result on the colloidal gold test combined with a WBC count > 9.075 × 10^9^/L was 84.9%. These findings suggest that a decision tree model can significantly increase the PPV and NPV for the rapid influenza diagnosis.

In this study, sore throat, cough, dizziness and headache, and myalgia and arthralgia were the four most common symptoms despite an incidence of only 53–67%, which suggested that many patients with the influenza-like disease do not have a typical respiratory tract or systemic symptoms. In this study, there were significant differences in the prevalence of cough, productive cough, runny nose, and chills between the positive and negative nucleic acid test result groups (*P* < 0.05). However, further analysis has shown that their diagnostic performance was low, with the PPV and NPV in the range of 56.8–62.6% and 50.7–55.6%, respectively. Clinical symptoms were not included in the final decision tree, suggesting that they are insufficient to differentiate between influenza and non-influenza disease in clinical practice.

The influenza colloidal gold test is widely used by many major medical institutions for influenza screening because it is rapid (results within 15–20 min), easy to use, does not require any special equipment, can be performed at the bedside, and all medical staff can be trained in its use. However, its sensitivity varies. In this study, the sensitivity, specificity, PPV, and NPV of the colloidal gold influenza A test was 52.9%, 74.2%, 72.4%, and 55.2%, respectively, and the corresponding values for the colloidal gold influenza B test were 20.3%, 92.5%, 77.8%, and 47.7%, respectively, which were consistent with those reported in a meta-analysis by Chartrand et al.^10^. This meta-analysis included 159 studies and evaluated 26 rapid influenza tests, and found that the sensitivity and specificity of rapid tests was 62.3% (confidence interval [CI] 57.9–66.6%) and 98.2% (CI 97.5–98.7%), respectively. In addition, the overall sensitivity of these tests in adults was 53.9% (CI 47.9–59.8%); for influenza A and B, the sensitivity values were 64.6% (CI 59.0–70.1%) and 52.2% (CI 45.0–59.3%), respectively. This shows that the colloidal gold method alone is insufficient to discriminate between influenza and influenza-like disease. Studies have shown that the PPV range for a rapid influenza test kit is 30–80%^11,12^. Moreover, Eggers et al.^13^ reported that the sensitivity and NPV of a rapid influenza test were 40–50% and 55–56%, respectively.

In this study, the proportion of patients who underwent routine blood tests, colloidal gold tests, and chest X-rays was 94%, 82%, and 16%, respectively. This shows that, at the early stages of the disease, routine blood tests and influenza colloidal gold tests are commonly used. In this study, influenza patients with a positive nucleic acid test result had a lower WBC count and neutrophil percentage than the patients with a negative nucleic acid test result, whose lymphocyte and monocyte percentage were higher, and these differences are statistically significant (*P* < 0.05). A WBC count < 9.075 × 10^9^/L emerged as an important cutoff point. Among patients with a positive nucleic acid test result, 10% of patients had elevated WBC count and 28% had an elevated neutrophil count. It is plausible that some of the influenza patients included in this study had a concomitant bacterial infection.

The decision tree model in our study found that the monocyte percentage range is relevant to rapid influenza diagnosis. Patients with a monocyte percentage within 10.95–12.55% were more likely to have influenza, whereas the PPV of this parameter decreased at a monocyte percentage >12.55%. This finding can be explained given the growing understanding of the immune function of monocytes. Monocytes are critical regulators of the innate systemic inflammatory response^14^, and their levels tend to be elevated in severe infections. Aegerter et al.^15^ reported that monocytes play an important role in influenza. Alveolar macrophages originate from monocytes and have antibacterial effects. Moreover, influenza viruses enter the cytoplasm through receptor-mediated endocytosis and promote monocytes/macrophages to secrete inflammatory cytokines and chemokines (e.g., tumor necrosis factor-alpha, interleukin 1 [IL-1], IL-4, IL-6, interferon-gamma, etc.) and to induce immune responses and phagocytosis in lymphocytes, while removing damaged or senescent cells. Therefore, monocytes play an important role in antiviral immunity.

Nevertheless, monocyte count elevation has a complex function in influenza patients. A review of the contribution of lung macrophages and monocytes to influenza pathogenesis showed that lung macrophages have catabolic and immunosuppressive functions^16^. During the acute phase of inflammation, classical lung monocytes, and monocyte-derived dendritic cells have displayed proinflammatory function. Coates et al.^17^ reported that excessive inflammatory responses due to increased monocyte aggregation in the lungs of young mice cause severe influenza instead of increasing the capacity to control viral replication. This study delivered insights into severe influenza in adolescents, specifically, on the contribution of monocytes to secondary acute lung injury in children with influenza. Other studies worldwide have shown that monocytes/macrophages act as a repository, although certain viruses use these cells for productive replication^18^. Monocyte levels are frequently elevated in mycoplasma infection^19^, and monocytes play a crucial role in Human enterovirus 71 (EV71) infection^20^.

Significantly elevated monocyte levels may represent atypical lymphocytes. In influenza patients, the immune response of lymphocytes induces T-lymphocyte activation, with the resultant production of atypical lymphocytes, which are larger and have large nuclei and loose chromatin. Instruments used for hematological analysis tend to recognize these atypical lymphocytes as monocytes, which results in a false increase in monocyte percentage. In future research, the manual verification of routine blood test results from patients with significant monocyte percentage elevation can be used to determine whether these additional monocytes are atypical lymphocytes. However, significant monocyte count elevation may suggest the presence of non-influenza infections, and in such circumstances, testing for other respiratory pathogens can be performed.

In China’s primary care system, people with flu-like symptoms are routinely given blood routine examinations when they go to the clinic. Further pathogenic microorganisms tests depend on whether the primary care institution is equipped with relevant technology, as well as the patient’s willingness, payment method, and suggestion from the clinician. Patients attending primary care facilities often seek to be diagnosed and treated as soon as possible. Real-time PCR (RT-PCR), which generally results within 4–6 h, is ideal for influenza detection in primary care. However, the technology and conditions to carry out RT-PCR are usually not available in primary health care facilities. Some secondary and tertiary general hospitals in China can carry out RT-PCR detection. But due to the relatively complex technology, the actual detection efficiency is low, and the results need to be waited for 3–10 days. The CDC in some areas can offer free nucleic acid testing for influenza viruses, with a wait of 7–14 days for results. These conditions affected the timeliness of diagnosis, which could increase the likelihood of blind treatment with antibiotics. This phenomenon may be prevalent in primary care systems in the most underdeveloped regions of the world.

In addition, the high cost limits the use of RT-PCR for influenza diagnosis in primary care settings in China. Through interviews with other hospitals in China and employees of pharmaceutical companies in China and the United States, the author has learned about the prices of colloidal gold and RT-PCR technology in China, the United States, and Europe. Viral nucleic acid testing in China costs about 300 to 700 Chinese Yuan (CNY), or about $46.6–108.7 USD. The cost is comparable to the ~$96–100 USD in the United States. In Europe, it’s about $194. But for Chinese patients with lower income levels, the burden of this cost may be greater. The colloidal gold method requires a simpler test that can be performed in a primary care setting, and results can be obtained in as little as 20 min. In China, this method is also much cheaper, priced at 119 CNY, or about $18.50 USD. It’s about 17–40% of the cost of RT-PCR. The cost for colloidal gold is $28–33 (28–34% of PCR) in the US, $73 (38% of PCR cost) in Europe.

The limitations of this study include the small sample size and selection bias. The minimum age of patients that presented at our outpatient fever clinic was 16 years. Hence, in this study, >70% of patients were young adults; 50% of patients sought medical attention on day 1 of their disease course, and two-thirds had a moderate-grade fever (38–39 °C). The proportion of patients with underlying disease, pregnancy, or older age was low (7.4%). None of the participants had received vaccination against influenza.

The decision tree analysis proposed in this study enables an easier understanding of the classification rules and presents a new influenza diagnosis approach. This approach improved the predictive performance of a single test, suggesting that it can guide antibiotic prescription in clinical practice. Of the 520 patients with influenza-like illness in this study, 105 (20%) had a negative colloidal gold test result and WBC count >9.0755 × 10^9^/L. These patients were considered “non-influenza” cases, which may involve bacterial infection, for which antibiotics might be recommended. In contrast, patients with a positive colloidal gold test result and monocyte percentage within 10.95–12.55% were considered likely to have influenza. The PPV of these parameters was 89.9%, suggesting that 69 (13%) patients did not require antibiotic treatment. In this study sample (*n* = 520), there was an evidence-based rationale for antibiotic use after a simple and rapid test among one-third of the patients.

Future prospective studies should test the feasibility of clinical use of the proposed decision tree model. In addition, studies that involve a larger sample size should be conducted to verify the diagnostic accuracy and stability of the model. For patients with negative nucleic acid test results, in whom the pathogen is unknown, high-throughput sequencing can be combined with differences in outcomes after treatment to determine the type of pathogen involved. However, further research is required to optimize the decision tree.

## Methods

All study participants provided informed consent, and the study design was approved by the Peking University Third Hospital Medical Science Research Ethics Committee 2017 (295–02). All subjects were fully informed and signed written informed consent prior to take part in the study.

This study prospectively enrolled patients who presented with fever at an outpatient clinic in Peking University Third Hospital during three influenza seasons (December 2017 to March 2018, December 2018 to March 2019, and December 2019 to January 2020). The inclusion criteria were as follows: fever ≥ 38 °C, cough, sore throat, and disease course of ≤3 days. This study excluded: (1) individuals who were mentally incapable or unable to understand the study requirements; (2) expected to have poor compliance; (3) pregnant or lactating women; and (4) other reasons that were considered inappropriate for participation in this study. As a national influenza surveillance outpost hospital, the mission was to collect nasopharyngeal swabs from 20 patients with influenza-like illness each week (ten patients on Mondays and Wednesdays, respectively). The study used data from this group of people. A total of 700 patients fulfilled the study eligibility criteria. We subsequently excluded 12 patients due to missing clinical data. Finally, 346 and 342 patients from the 2017–2018 and 2018–2019 flu season, were selected, respectively, for study inclusion and provided a total study sample of 688 patients. However, 109 patients with body temperature < 38 °C and 59 patients with disease course of >3 days/unclear information in their medical records were excluded. The final analysis dataset included 520 patients. In the 2019–2020 influenza season, only 160 cases were collected within 2 months due to the coronavirus disease epidemic. Among these cases, four were excluded due to missing clinical data. Thus, 156 cases were included for the 2019–2020 influenza season.

### Laboratory methods and clinical data

Nasopharyngeal swabs were prospectively collected and tested. The colloidal gold method was applied immunochromatography and a double-antibody sandwich to detect influenza A/B antigens by influenza A/B viral antigen detection kit (Guangzhou Wongfo Biotech Co., Ltd, Guangzhou, China). Preservative solution (80 µL) containing the dissolved sample was added to the well in the test card, and chromatography was performed after 15–20 min to detect the influenza antigens.

The nucleic acid test was used for a definitive diagnosis of influenza. The specimens were delivered to the laboratory of Beijing Haidian District Center for Disease Control and Prevention following a standard institutional protocol used at the study center, which is a national sentinel surveillance hospital. The influenza A/B nucleic acid assay kit, H1N1 (2009) influenza A/seasonal influenza H3 nucleic acid assay kit and the Victoria/Yamagata (BV/BY) influenza B nucleic acid test kit (Jiangsu Bioperfectus Technologies Co., Ltd) were used and assayed for each sample on the ABI 7500fast real-time quantitative PCR system.

Data on demographics (i.e., age and sex), clinical presentation (i.e., body temperature, °C; disease course, days; cough, sputum, dyspnea, runny nose, diarrhea, dizziness, headache, myalgia/arthralgia, fatigue, and chills) were collated. Medical history (i.e., underlying disease, pregnancy, contact history, influenza vaccination, and high-risk population), routine blood test results (i.e., white blood cell count × 10^9^/L, hemoglobin g/L, platelet count × 10^12^/L, lymphocyte %, and monocyte %), and influenza colloidal gold test were included for analysis.

### Statistical analysis

SPSS version 17.0 (Chicago: SPSS Inc.) was used for statistical analysis. Quantitative data are shown as mean and standard deviation, and qualitative data are presented as count and percentage. The *t* test or chi-square test was used for intergroup comparisons of positive/negative results of the nucleic acid test. The correlation between general clinical characteristics and nucleic acid test results was examined. Clinical characteristics were evaluated as independent variables, and CRT was used to construct a decision tree. The CRT method involves dividing the population into several homogeneous subpopulations with specific characteristics. Thus, the derived subgroups have a high degree of internal consistency and a similar degree of internal variation/impurity, which can be achieved by applying the prediction error minimization or a binary method^21^. The 2017–2018 and 2018–2019 influenza season data were used to establish the decision tree, while the 2019–2020 influenza season data were used for verification.

### Reporting summary

Further information on research design is available in the Nature Research Reporting Summary linked to this article.

## Supplementary information

Reporting Summary

## Data Availability

The data will be made available to others on reasonable requests to the corresponding author.

## References

[1] World Health Organization. *Influenza (Seasonal)*. http://www.who.int/news-room/fact-sheets/detail/influenza-(seasonal). (WHO, 2018).

[2] Iuliano ADanielle (2018). “Estimates of global seasonal influenza-associated respiratory mortality: a modelling study.”. Lancet.

[3] Craddock K (2020). “The impact of educational interventions on antibiotic prescribing for acute upper respiratory tract infections in the ambulatory care setting: a quasi‐experimental study. J. Am. Coll. Clin. Pharm..

[4] Havers FP (2018). “Outpatient antibiotic prescribing for acute respiratory infections during influenza seasons,”. JAMA Netw. Open.

[5] Taymaz T (2018). Significance of the detection of influenza and other respiratory viruses for antibiotic stewardship: lessons from the post-pandemic period. Int. J. Infect. Dis..

[6] Durkin MJ (2018). Outpatient antibiotic prescription trends in the United States: a national cohort study. Infect. Control Hosp. Epidemiol..

[7] Eboigbodin K (2016). Reverse transcription strand invasion based amplification (RT-SIBA): a method for rapid detection of influenza A and B. Appl. Microbiol. Biotechnol..

[8] Nolte FS, Gauld L, Barrett SB (2016). Direct comparison of alere I and Cobas liat influenza A and B tests for rapid detection of influenza virus infection. J. Clin. Microbiol..

[9] Laxminarayan R (2013). Antibiotic resistance—the need for global solutions. Lancet Infect. Dis..

[10] Chartrand C (2012). Accuracy of rapid influenza diagnostic tests: a meta-analysis. Ann. Intern Med.

[11] Zazueta-Garcia R (2014). Effectiveness of two rapid influenza tests in comparison to reverse transcription-PCR for influenza A diagnosis. J. Infect. Dev. Ctries.

[12] Ndegwa LK (2017). Evaluation of the point-of-care Becton Dickinson Veritor™ rapid influenza diagnostic test in Kenya, 2013-2014. BMC Infect. Dis..

[13] Eggers M, Enders M, Ladwig ET (2015). Evaluation of the Becton Dickinson rapid influenza diagnostic tests in outpatients in Germany during seven influenza seasons. PLoS ONE.

[14] Krychtiuk KA (2016). Monocyte subset distribution is associated with mortality in critically ill patients. Thromb. Haemost..

[15] Aegerter H (2020). Influenza-induced monocyte-derived alveolar macrophages confer prolonged antibacterial protection. Nat. Immunol..

[16] Duan MB, Hibbs ML, Chen WS (2017). The contributions of lung macrophage and monocyte heterogeneity to influenza pathogenesis. Immunol. Cell Biol..

[17] Coates BM (2018). Inflammatory monocytes drive influenza A virus-mediated lung injury in juvenile mice. J. Immunol..

[18] Nikitina E (2018). Monocytes and macrophages as viral targets and reservoirs. Int. J. Mol. Sci..

[19] Wang Z (2019). Monocyte subsets study in children with *Mycoplasma pneumoniae* pneumonia. Immunol. Res.

[20] Wongsa A (2019). Replication and cytokine profiles of different subgenotypes of enterovirus 71 isolated from Thai patients in peripheral blood mononuclear cells. Micro. Pathog..

[21] Wray CM, Byers AL (2020). Methodological progress note: classification and regression tree analysis. J. Hosp. Med..

