# Supplementary material for: Influenza colloidal gold method and blood routine tests combination for rapid diagnosis of influenza: a decision tree-based analysis
Source: NPJ Prim Care Respir Med. 2021 Jul 15;31:39. doi: 10.1038/s41533-021-00251-x (PMC8282862; doi:10.1038/s41533-021-00251-x)
Supplement: Supplementary file 1 — Reporting Summary [file 41533_2021_251_MOESM1_ESM.pdf]

nature research

Corresponding author(s): Xiaoguang Li, Jing Chen, Fei Lin, Wei Wang, Jie Xu, Nan Li

Last updated by author(s): May 14, 2021

Reporting Summary

Nature Research wishes to improve the reproducibility of the work that we publish. This form provides structure for consistency and transparency in reporting. For further information on Nature Research policies, see our Editorial Policies and the Editorial Policy Checklist.

Statistics

For all statistical analyses, confirm that the following items are present in the figure legend, table legend, main text, or Methods section.

n/a	Confirmed	
☐	☒	The exact sample size (n) for each experimental group/condition, given as a discrete number and unit of measurement
☐	☒	A statement on whether measurements were taken from distinct samples or whether the same sample was measured repeatedly
☐	☒	The statistical test(s) used AND whether they are one- or two-sided<br>*Only common tests should be described solely by name; describe more complex techniques in the Methods section.*
☒	☐	A description of all covariates tested
☒	☐	A description of any assumptions or corrections, such as tests of normality and adjustment for multiple comparisons
☐	☒	A full description of the statistical parameters including central tendency (e.g. means) or other basic estimates (e.g. regression coefficient) AND variation (e.g. standard deviation) or associated estimates of uncertainty (e.g. confidence intervals)
☐	☒	For null hypothesis testing, the test statistic (e.g. F, t, r) with confidence intervals, effect sizes, degrees of freedom and P value noted<br>*Give P values as exact values whenever suitable.*
☒	☐	For Bayesian analysis, information on the choice of priors and Markov chain Monte Carlo settings
☐	☒	For hierarchical and complex designs, identification of the appropriate level for tests and full reporting of outcomes
☒	☐	Estimates of effect sizes (e.g. Cohen's d, Pearson's r), indicating how they were calculated

Our web collection on statistics for biologists contains articles on many of the points above.

Software and code

Policy information about availability of computer code

Data collection	In this study, printed case report forms were used for data collection, and Microsoft Excel was used for data entry.
Data analysis	SPSS 26.0 (IBM Corp.) was used for data analysis.

For manuscripts utilizing custom algorithms or software that are central to the research but not yet described in published literature, software must be made available to editors and reviewers. We strongly encourage code deposition in a community repository (e.g. GitHub). See the Nature Research guidelines for submitting code & software for further information.

Data

Policy information about availability of data

All manuscripts must include a data availability statement. This statement should provide the following information, where applicable:

- Accession codes, unique identifiers, or web links for publicly available datasets
- A list of figures that have associated raw data
- A description of any restrictions on data availability

The decision tree analysis data that support the findings of this study are available on request from the corresponding author (N. L.). The data are not publicly available due to them containing information that could compromise research participant privacy or consent. The informed consent process does not require patients to consent to make the data from them available to the public.

Field-specific reporting

Please select the one below that is the best fit for your research. If you are not sure, read the appropriate sections before making your selection.

☒ Life sciences ☐ Behavioural & social sciences ☐ Ecological, evolutionary & environmental sciences

For a reference copy of the document with all sections, see nature.com/documents/nr-reporting-summary-flat.pdf

Life sciences study design

All studies must disclose on these points even when the disclosure is negative.

Sample size	This study prospectively enrolled patients who presented with fever at an outpatient clinic in Peking University Third Hospital during three influenza seasons (December 2017 to March 2018, December 2018 to March 2019, and December 2019 to January 2020). As a national influenza surveillance outpost hospital, the mission was to collect nasopharyngeal swabs from 20 patients with influenza-like illness each week (10 patients on Mondays and Wednesdays, respectively). The study used data from this group of people. A total of 700 patients fulfilled the study eligibility criteria.
Data exclusions	We subsequently excluded 12 patients due to missing clinical data. Finally, 346 and 342 patients from the 2017–2018 and 2018–2019 flu season, were selected, respectively, for study inclusion and provided a total study sample of 688 patients. However, 109 patients with body temperature <38? and 59 patients with disease course of >3 days/unclear information in their medical records were excluded. The final analysis dataset included 520 patients.In the 2019–2020 influenza season, only 160 cases were collected within 2 months due to the coronavirus disease epidemic. Among these cases, four were excluded due to missing clinical data. Thus, 156 cases were included for the 2019–2020 influenza season.
Replication	In order to reproduce and verify whether routine blood data combined with colloidal gold test results can effectively diagnose influenza, part of the data was used in this study to establish a diagnostic model and the subsequent data were used for external validation. The 2017–2018 and 2018–2019 influenza season data were used to establish the decision tree, while the 2019–2020 influenza season data were used for verification.
Randomization	This study was a diagnostic test, so all subjects received both different diagnostic methods simultaneously. Randomized allocation of interventions was not required in this study.
Blinding	This study is an observational study, so it does not involve the concealment of random assignment scheme. All methods of clinical examination were measured before the patient's clinical outcome was known. All patients underwent the same clinical tests.

Reporting for specific materials, systems and methods

We require information from authors about some types of materials, experimental systems and methods used in many studies. Here, indicate whether each material, system or method listed is relevant to your study. If you are not sure if a list item applies to your research, read the appropriate section before selecting a response.

Materials & experimental systems

n/a	Involved in the study	
☒	☐	Antibodies
☒	☐	Eukaryotic cell lines
☒	☐	Palaeontology and archaeology
☒	☐	Animals and other organisms
☐	☒	Human research participants
☐	☒	Clinical data
☒	☐	Dual use research of concern

Methods

n/a	Involved in the study	
☒	☐	ChIP-seq
☒	☐	Flow cytometry
☒	☐	MRI-based neuroimaging

Human research participants

Policy information about studies involving human research participants

Population characteristics	Table 1 presents the clinical presentation characteristics of 520 patients with influenza-like illness (fever ≥38?, cough, sore throat, and disease course of ≤3 days). In 520 patients, mean age was 34.82 ± 14.06 years and 249 was male. 271 with "positive"nucleic acid test results and 249 patients with "negative" nucleic acid test results. Among the 271 patients with positive nucleic acid test results, there were 116 cases of H1N1 (2009) influenza A, 55 cases of seasonal influenza H3, 69 cases of Yamagata (BY) influenza B, and 31 cases of Victoria (BV) influenza B.
Recruitment	This study prospectively enrolled patients who presented with fever at an outpatient clinic in Peking University Third Hospital during three influenza seasons (December 2017 to March 2018, December 2018 to March 2019, and December 2019 to January 2020). As a national influenza surveillance outpost hospital, the mission was to collect nasopharyngeal swabs from 20 patients with influenza-like illness each week (10 patients on Mondays and Wednesdays, respectively). The study used data

from this group of people.
The limitations of this study include the small sample size and selection bias. The minimum age of patients that presented at our outpatient fever clinic was 16 years. Hence, in this study, more than 70% of patients were young adults. The proportion of patients with underlying disease, pregnancy, or older age was low (7.4%). None of the participants had received vaccination against influenza.

Ethics oversight	This study design was approved by the Peking University Third Hospital Medical Science Research Ethics Committee 2017 (295-02). All participants were fully informed and signed written informed consent prior to take part in the study. All participants provided informed consent.

Note that full information on the approval of the study protocol must also be provided in the manuscript.

Clinical data

Policy information about clinical studies

All manuscripts should comply with the ICMJE guidelines for publication of clinical research and a completed CONSORT checklist must be included with all submissions.

Clinical trial registration	Registered on Chinese Clinical Trial Registry (ChiCTR), one of the primary registries in the WHO registry network. The ID is: ChiCTR1900022708
Study protocol	This study is an observational study. The research protocols have not been published. However, the research design and protocal have been completed before the application of ethics and formal implementation.
Data collection	All data were collected at an outpatient clinic in Peking University Third Hospital, Beijing, China. Patients with fever meeting inclusion but not exclusion criteria were continuously included during three influenza seasons (December 2017 to March 2018, December 2018 to March 2019, and December 2019 to January 2020).
Outcomes	This study was a diagnostic test. The target disease for diagnosis is influenza. Therefore, the primary outcome can be considered as the diagnosis of influenza. The pre-defined outcome measures are the real-time quantitative PCR. The influenza A/B nucleic acid assay kit, H1N1 (2009) influenza A/seasonal influenza H3 nucleic acid assay kit, and the Victoria/Yamagata (BV/BY) influenza B nucleic acid test kit (Jiangsu Bioperfectus Technologies Co.,Ltd), all samples were assayed on the ABI 7500fast real-time quantitative PCR system. This test is currently the gold standard for influenza testing.
